# Supplementary material for: HPV E6 regulates therapy responses in oropharyngeal cancer by repressing the PGC-1α/ERRα axis
Source: JCI Insight. 2022 Sep 22;7(18):e159600. doi: 10.1172/jci.insight.159600 (PMC9675449; doi:10.1172/jci.insight.159600)
Supplement: Supplemental data [file jciinsight-7-159600-s171.pdf]

INDEX

Supplemental Figure 1. Characterization of mitochondrial function and cisplatin response in HPV+ PDXs and cell lines

Supplemental Figure 2. Lack of associations between mitochondrial mass and levels of other HPV oncogenic transcripts in TCGA and the PDX panel

Supplemental Figure 3. Metabolic effects of increasing fl-E6 mRNA expression on VU147T cells, SCC154 cells, and N-tert/E7 keratinocytes

Supplemental Figure 4. Effect of increased fl-E6 on treatment sensitization of VU147T and SCC154 cells

Supplemental Figure 5. PGC-1α overcomes fl-E6-mediated reduction in expression of mitochondrial components

Supplemental Figure 6. Effects of increased fl-E6 expression on the PGC-1α/ERRα axis in cancer cell lines, N-tert/E7 keratinocytes, and HEK293 cells

Supplemental Table 1. Patient characteristics in TCGA, JHU, and VU cohorts

Supplemental Table 2. Primers used for qPCR analysis

Supplemental Table 3. Antibodies used in the study

Supplemental Table 4. Plasmids used in the study

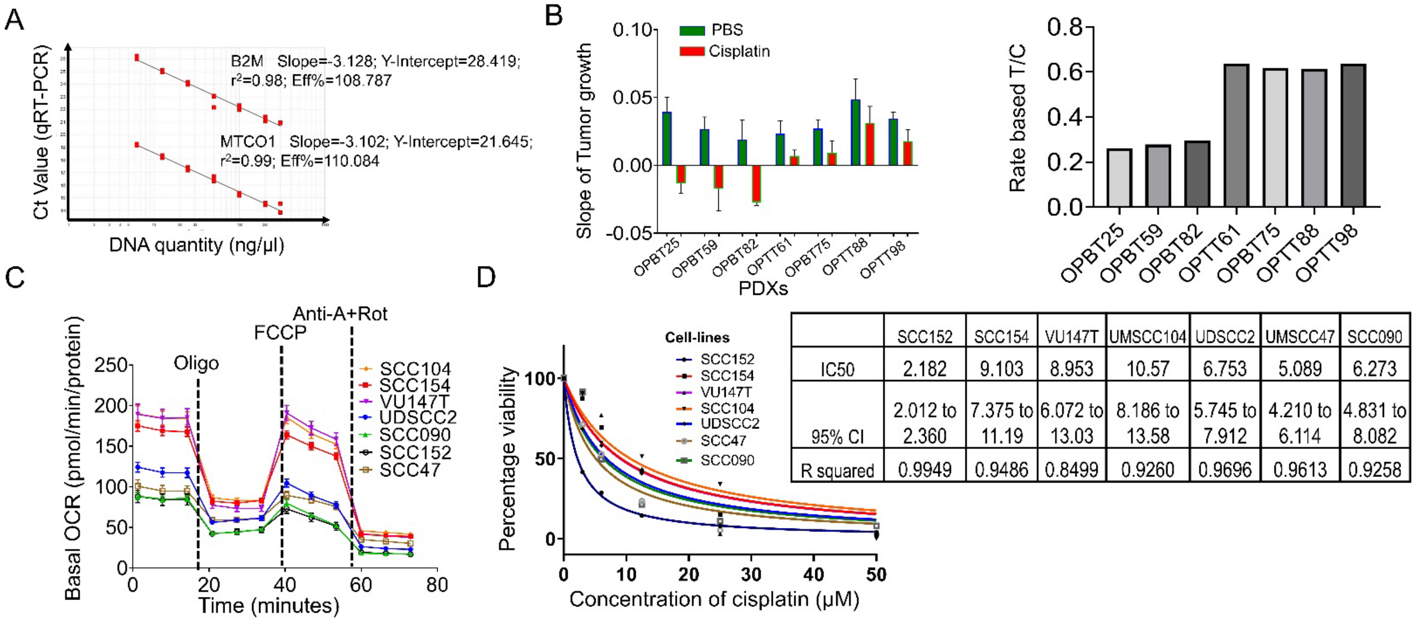

	SCC152	SCC154	VU147T	UMSCC104	UDSCC2	UMSCC47	SCC090
IC50	2.182	9.103	8.953	10.57	6.753	5.089	6.273
95% CI	2.012 to 2.360	7.375 to 11.19	6.072 to 13.03	8.186 to 13.58	5.745 to 7.912	4.210 to 6.114	4.831 to 8.082
R squared	0.9949	0.9486	0.8499	0.9260	0.9696	0.9613	0.9258

Supplemental Figure 1. Characterization of mitochondrial function and cisplatin response in HPV+ PDXs and cell lines. (A) Standard curves used to determine *MTCO1/B2M* ratio by DNA qPCR. **(B)** Tumor growth rate slopes used to calculate rate-based T/C value [25] for PDXs after intraperitoneal administration of PBS or Cisplatin 3 times/week for 2 weeks. At least 4 biological replicates were used for each condition, and tumor volumes were measured at times of cisplatin administration. **(C)** Seahorse Assay for cell line panel showing normalized OCR profile after addition of oligomycin (Oligo, 0.001 mg/ml), carbonyl cyanide p-trifluoro-methoxyphenyl hydrazone (FCCP, 2.5 µM), and antimycin + rotenone (Anti-A+Rot, 2 µM each). **(D)** Dose response curve for cisplatin vs. normalized cell viability by WST assay after 48 hours treatment. IC50 with 95% confidence intervals (CI) and R^2 were obtained from simple logarithmic regression. Data points represent mean ± SEM.

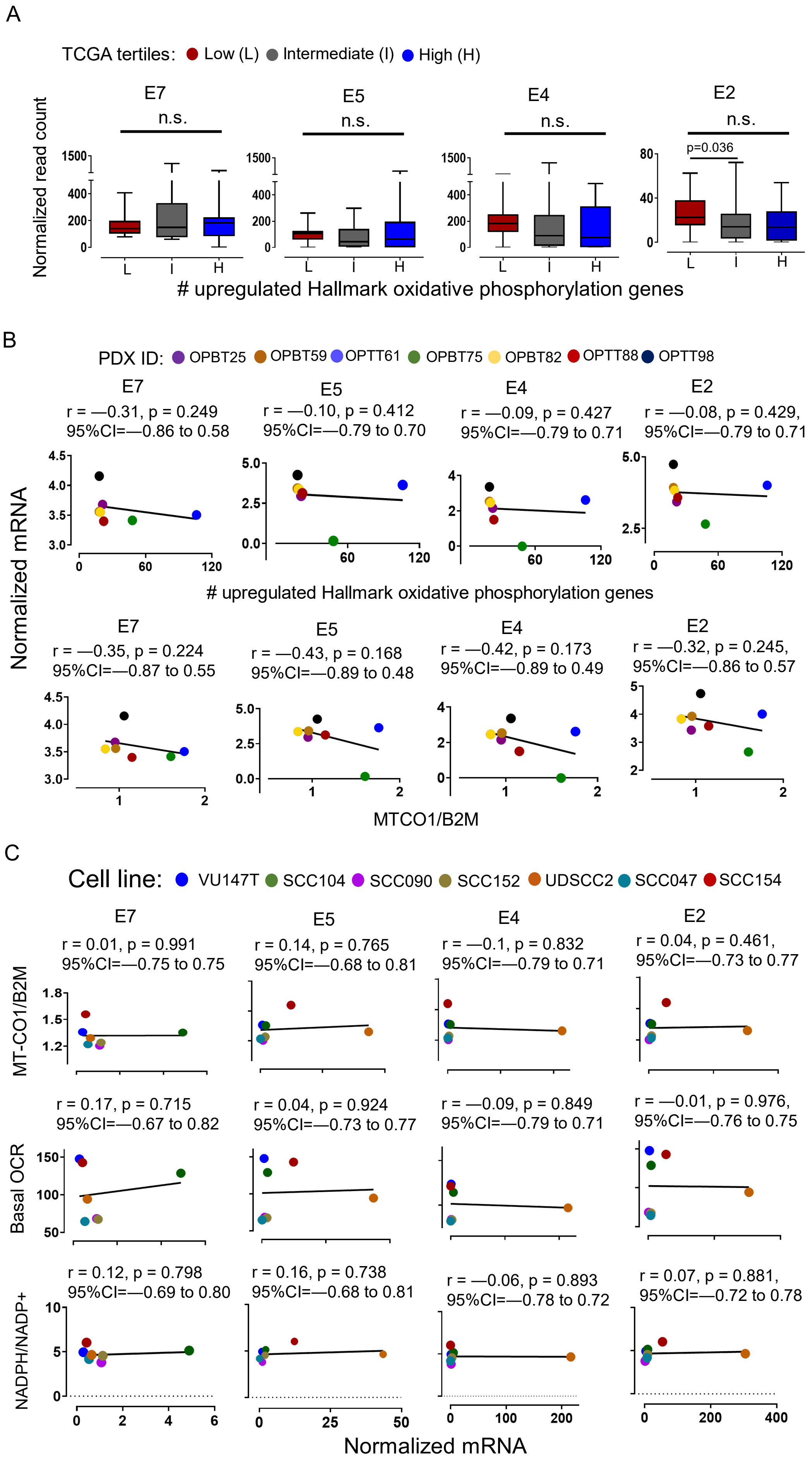
A
TCGA tertiles: Low (L) Intermediate (I) High (H)
E7 E5 E4 E2
n.s.
p=0.036
Normalized read count
upregulated Hallmark oxidative phosphorylation genes
B
PDX ID: OPBT25 OPBT59 OPTT61 OPBT75 OPBT82 OPTT88 OPTT98
r = —0.31, p = 0.249
95%CI=—0.86 to 0.58
r = —0.10, p = 0.412
95%CI=—0.79 to 0.70
r = —0.09, p = 0.427
95%CI=—0.79 to 0.71
r = —0.08, p = 0.429,
95%CI=—0.79 to 0.71
Normalized mRNA
# upregulated Hallmark oxidative phosphorylation genes
r = —0.35, p = 0.224
95%CI=—0.87 to 0.55
r = —0.43, p = 0.168
95%CI=—0.89 to 0.48
r = —0.42, p = 0.173
95%CI=—0.89 to 0.49
r = —0.32, p = 0.245,
95%CI=—0.86 to 0.57
MTCO1/B2M
C
Cell line: VU147T SCC104 SCC090 SCC152 UDSCC2 SCC047 SCC154
r = 0.01, p = 0.991
95%CI=—0.75 to 0.75
r = 0.14, p = 0.765
95%CI=—0.68 to 0.81
r = —0.1, p = 0.832
95%CI=—0.79 to 0.71
r = 0.04, p = 0.461,
95%CI=—0.73 to 0.77
MT-CO1/B2M
r = 0.17, p = 0.715
95%CI=—0.67 to 0.82
r = 0.04, p = 0.924
95%CI=—0.73 to 0.77
r = —0.09, p = 0.849
95%CI=—0.79 to 0.71
r = —0.01, p = 0.976,
95%CI=—0.76 to 0.75
Basal OCR
r = 0.12, p = 0.798
95%CI=—0.69 to 0.80
r = 0.16, p = 0.738
95%CI=—0.68 to 0.81
r = —0.06, p = 0.893
95%CI=—0.78 to 0.72
r = 0.07, p = 0.881,
95%CI=—0.72 to 0.78
NADPH/NADP+
Normalized mRNA

Supplemental Figure 2. Lack of associations between mitochondrial mass and levels of other HPV oncogenic transcripts in TCGA and the PDX panel. (A) RNAseq evaluating E7, E5, E4, and E2, for differential expression among tertiles of Hallmark Oxidative Phosphorylation gene expression in TCGA. p values calculated by Mann Whitney Test. **(B)** Scatter plots for the PDXs evaluating the same HPV transcript levels vs. number of up-regulated Hallmark Oxidative Phosphorylation transcripts (top) and mitochondrial mass (*MT-CO1/B2M* by DNA qPCR) (bottom). **(C)** Scatter plots for HPV+ cell lines evaluating the same HPV transcript levels vs. mitochondrial mass (MT-CO1/B2M DNA qPCR, above), basal OCR (middle) measured by Seahorse Assay and vs. NADPH/NADP+ (below) measured by enzyme cycling-based colorimetric assay. Pearson correlation coefficients were used to calculate r values with confidence intervals, p values determined by t-distribution.

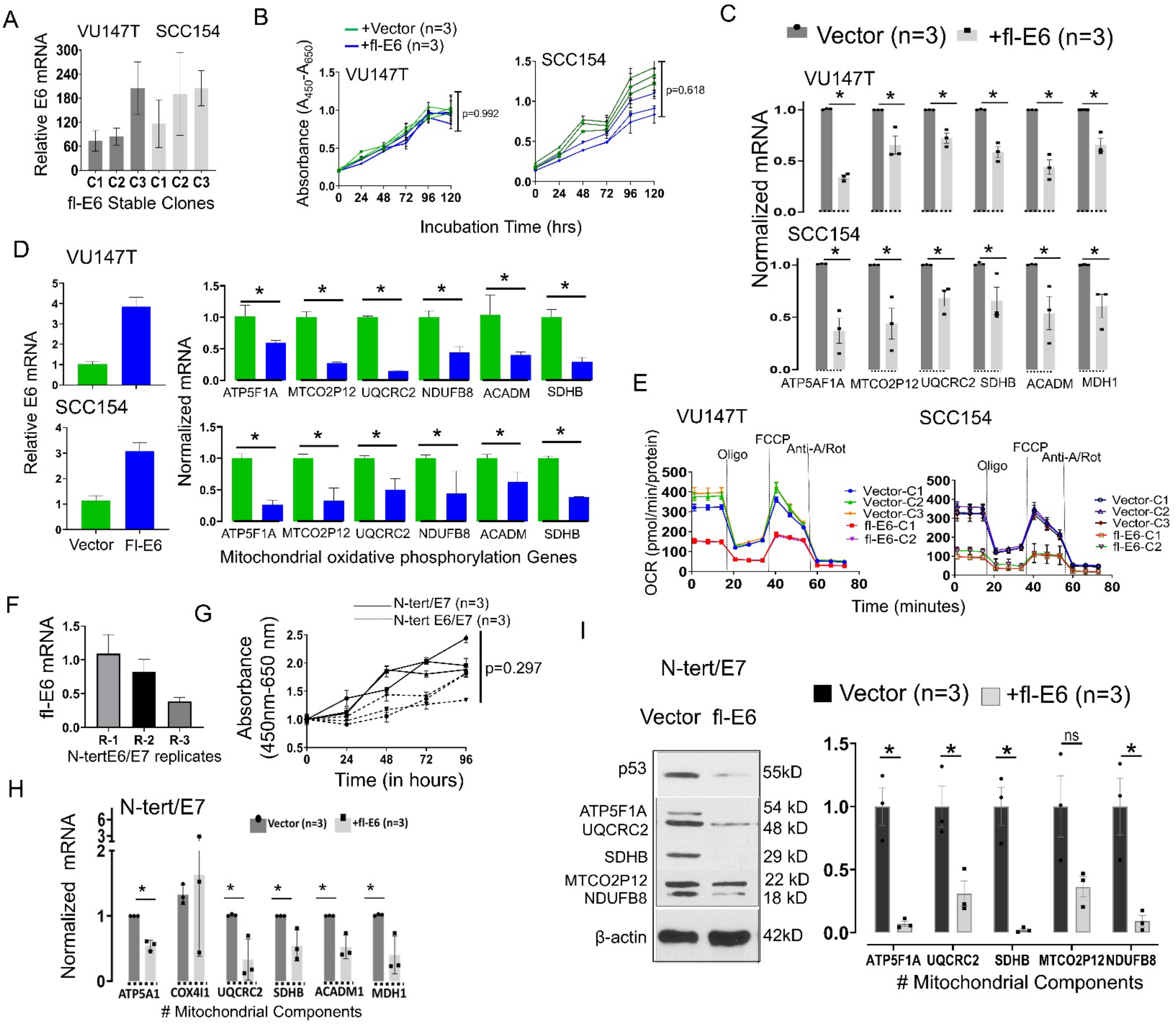

Supplemental Figure 3. Metabolic effects of increasing fl-E6 mRNA expression on VU147T cells, SCC154 cells, and N-tert/E7 keratinocytes. (A) RT-qPCR of E6 mRNA normalized to 18S in 3 lentiviral fl-E6 transfected clones and 3 vector control clones of SCC154 and VU147T. **(B)** Growth rate of SCC154 and VU147T upon stable fl-E6 expression by WST assay. p value is calculated using ANOVA. **(C)** RT-qPCR upon fl-E6 expression for 6 genes involved in oxidative phosphorylation, with 18S as internal control. **(D)** RT-qPCR upon transient fl-E6 over-expression for 6 genes involved in oxidative phosphorylation, with 18S as internal control. Bars represent mean ± SEM for three biologic replicates. p value calculated using unpaired t test. $^*p<0.05$. **(E)** Seahorse Assay showing effect of fl-E6 expression on normalized OCR profile after addition of oligomycin (Oligo, 0.001 mg/ml), carbonyl cyanide p-trifluoro-methoxyphenyl hydrazone (FCCP, 2.5 μM), and

antimycin + rotenone (Anti-A+Rot, 2 µM each). **(F)** RT-qPCR for fl-E6 mRNA normalized to 18S upon stable transfection of N-tert/E7 keratinocytes. **(G)** Effect of fl-E6 expression on growth rate of N-tert/E7 keratinocytes by WST assay, showing 3 biologic replicates per condition. p value by ANOVA. **(H)** RT-qPCR in N-tert/E7 keratinocytes +/- fl-E6 for 6 genes involved in oxidative phosphorylation normalized by 18S. **(I)** Representative western blot (right) and their densitometric quantification (left) of 3 biologic replicates of N-Tert/E7 cells+/-fl-E6 showing p53 and five electron transport chain components. Values represent mean ± SEM for three biologic replicates. p value by unpaired t test. *$p<0.05$.

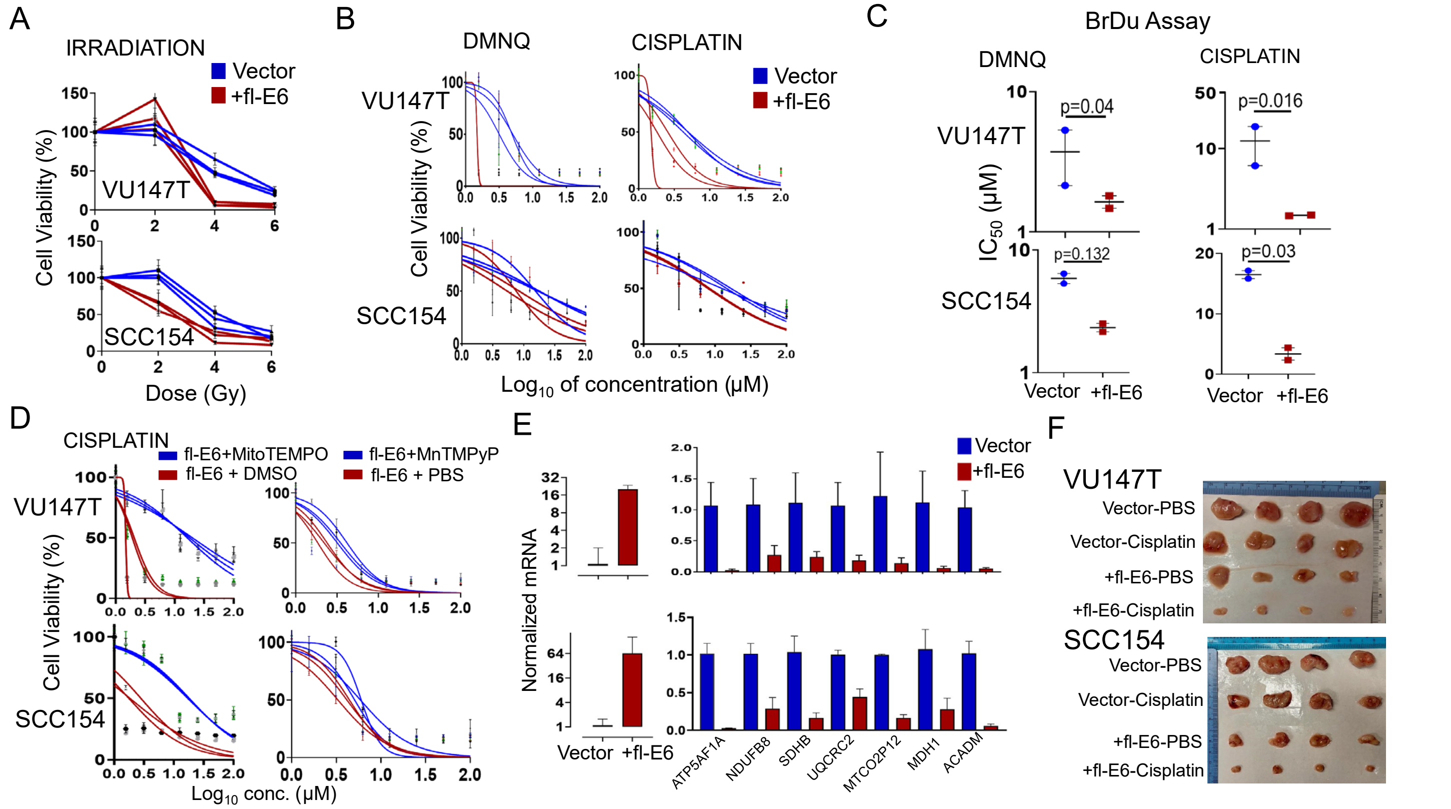

Supplemental Figure 4. Effect of increased fl-E6 on treatment sensitization of VU147T and SCC154 cells. *In vitro* IC_{50} were quantified by simple logistic regression. **(A)** % surviving cells 10 days after 2, 4, or 6 Gy radiation. **(B)** Dose responses to DMNQ (left) and cisplatin (right) based on % surviving cells after 48 hours of treatment using WST assay. **(C)** IC_{50} for DMNQ (left) and cisplatin (right) after 48 hours of treatment using BrDu colorimetric assay. **(D)** Dose responses to cisplatin +/- 10µM MitoTEMPO/DMSO or +/- 100µM MnTMPyP after 48 hours of treatment using BrDu colorimetric assay. Each point on the survival curve and dose response represents the mean surviving fraction from at least three replicates. **(E)** VU147T and SCC154 cells +/- stable fl-E6 expression was grown subcutaneously *in vivo* for 2 weeks. qRT-PCR normalized to 18S shows expression of fl-E6 and 7 genes involved in oxidative phosphorylation in resulting solid tumors. Bars represent mean ± SEM for two solid tumors per group. **(F)** Image of solid tumors used to quantify *in vivo* responses to cisplatin in Figure 4F.

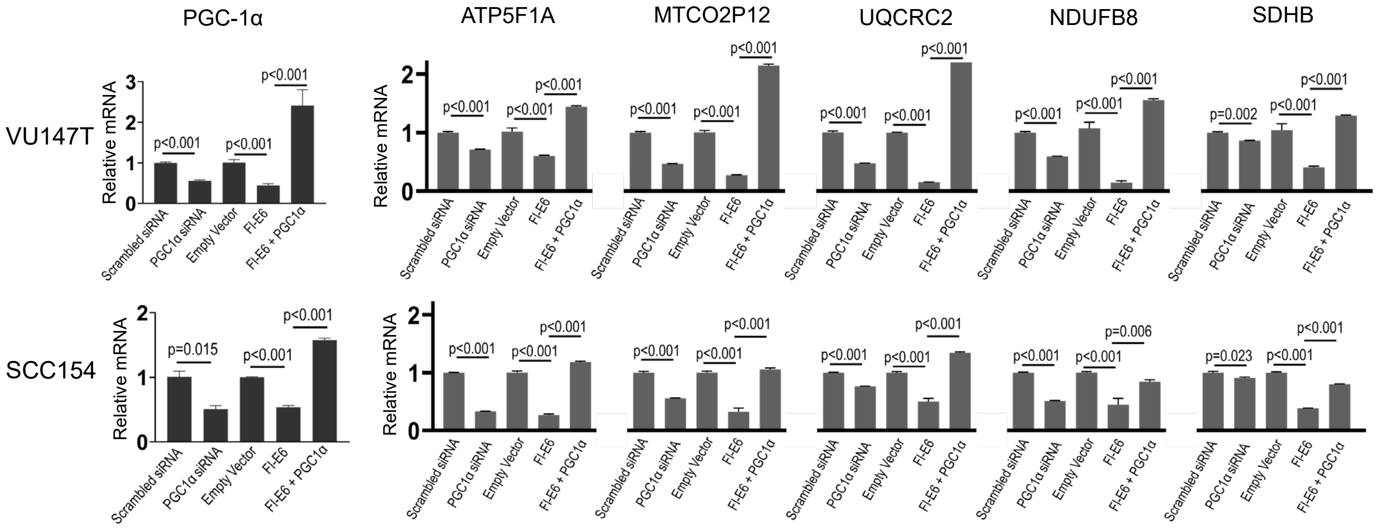

Supplemental Figure 5. **PGC-1α overcomes fl-E6-mediated reduction in expression of mitochondrial components.** RT-qPCR in VU147T and SCC154 cells for PGC1α (left) and mitochondrial components (right) after PGC-1α silencing and fl-E6 overexpression alone and in combination. 18S is used as the internal control. Bars represent mean ± SEM. p values are calculated using unpaired t tests.

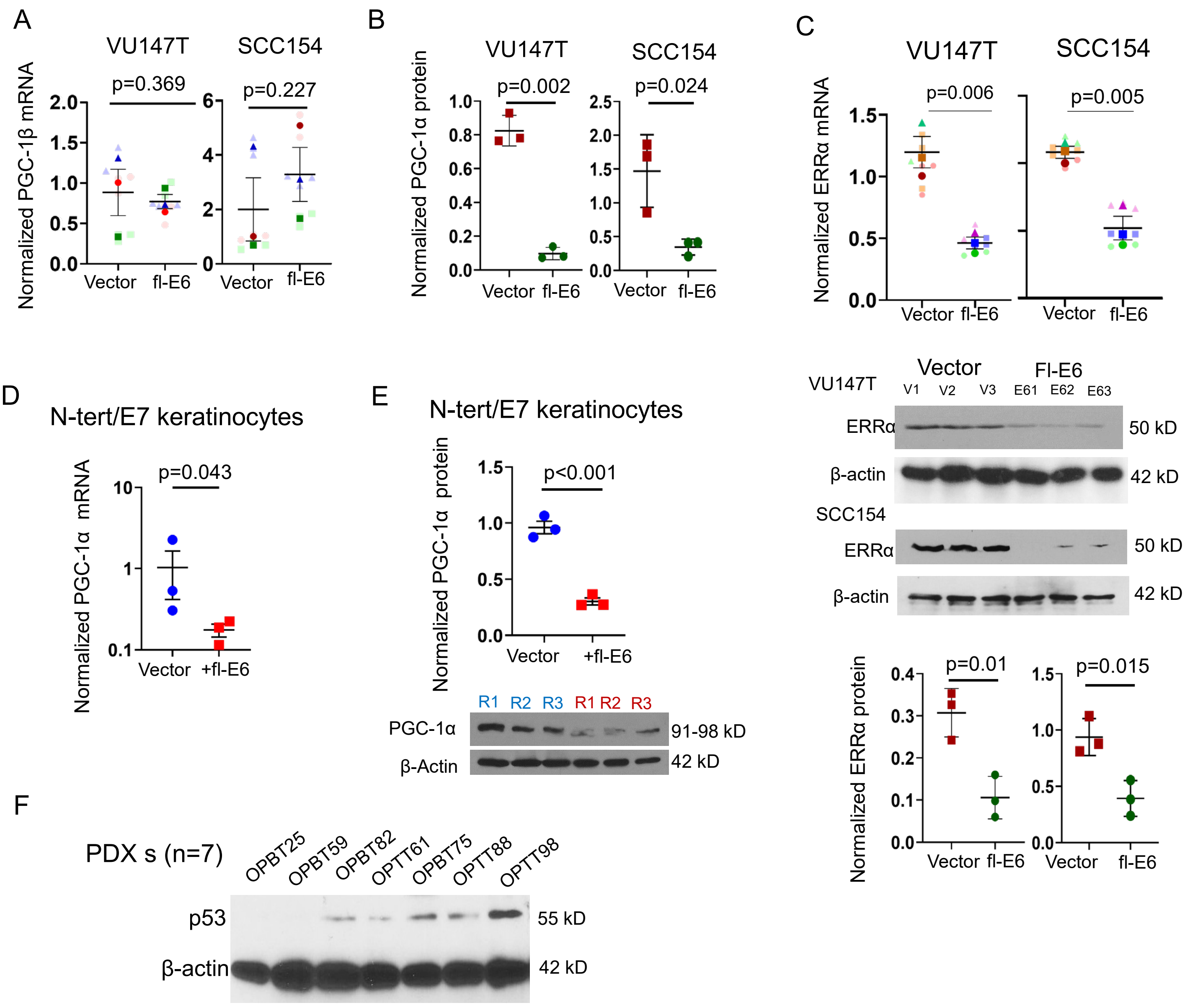

Supplemental Figure 6. Effects of increased fl-E6 expression on the PGC-1α/ERRα axis in cancer cell lines, N-tert/E7 keratinocytes, and HEK293 cells. (A) RT-qPCR of PGC-1β transcript normalized to 18S. **(B)** Western blot densitometry for PGC-1α normalized to β-actin. **(C)** RT-qPCR of ERRα transcript normalized to 18S (top) and western blot with densitometry for ERRα (bottom) normalized to β-actin in 3 lentiviral fl-E6 transfected clones and 3 vector control clones of SCC154 and VU147T. Plots represent mean ± SEM for 3 biologic replicates. p value calculated using unpaired t test. **(D)** RT-qPCR of PGC-1α transcript normalized to 18S. **(E)** Western blot of PGC-1α (left), with band densities (right) normalized to β-actin in 3 lentiviral fl-E6 transfected and 3 vector control replicates of N-tert/E7 keratinocytes. Plots represent mean ± SEM for the biologic replicates. p value calculated using unpaired t test. **(F)** Western blot of p53 and β-actin in PDX panel.

Supplemental Table 1. Patient characteristics in TCGA, JHU, and VU cohorts

Variable	Category	TCGA (n=53)	JHU (n=47)	VU (n=37)
**Median age (range)**		56 (35-77)	55 (35-75)	58 (52-62)
**Gender, N (%)**	Male	48 (90.6)	42 (89.4)	2 (5.4)
	Female	5 (9.4)	5 (10.6)	35 (94.6)
**Clinical T-stage†, N (%)**	Early (Tx/T0–2)	40 (75.5)	35 (74.5)	23 (62.2)
	Advanced (T3–4)	5 (9.4)	12 (25.5)	13 (35.1)
	Unknown	8 (15.1)	0 (0)	1 (2.7)
**Clinical N-stage†, N (%)**	N0	7 (13.2)	2 (4.2)	3 (8.1)
	N1	5 (9.4)	5 (10.6)	6 (16.2)
	N2a	2 (3.8)	10 (21.3)	5 (13.5)
	N2b	9 (16.9)	20 (42.6)	14 (37.8)
	N2c	0 (0)	2 (4.2)	8 (21.6)
	N3	1 (2)	2 (4.2)	0 (0)
	Unknown	29 (54.7)	6 (12.8)	0 (0)
**Clinical M-stage†, N (%)**	M0	14 (26.4)	47 (100)	35 (94.6)
	M1	0 (0)	0 (0)	0 (0)
	Unknown	39 (73.6)	0 (0)	2 (5.4)
**Clinical overall stage†, N (%)**	Early (I–II)	4 (7.5)	0 (0)	4 (10.8)
	Advanced (III–IV)	22 (41.5)	47 (100)	31 (83.8)
	Unknown	27 (51)	0 (0)	2 (5.4)
**Therapy, N (%)**	Surgery alone	3 (5.7)	2 (4.3)	0 (0)
	Surgery+Radiation	2 (3.8)	14 (29.8)	0 (0)
	Surgery+CRT	4 (7.6)	15 (31.9)	0 (0)
	Radiation alone	2 (3.8)	2 (4.3)	0 (0)
	CRT	13 (24.4)	13 (27.6)	37 (100)
	Incomplete data	29 (54.7)	1 (2.1)	0 (0)

†7th edition AJCC staging manual

CRT=chemoradiotherapy; NA=Not Available

Supplemental Table 2. Primers used for qPCR

Gene	Forward	Reverse
**MTCO2P12**	CAGGGTATTTAGCCTAGTTGGC	GCCGATCCATATAAGCTGGGA
**MDH1**	TGCTGTCATCAAGGCTCGAA	CTCCCTCTGGGGTTCCAAAC
**ACADM**	GACTGAGGAGCCATTGATGTG	CCGTTGGTTATCCACATCTTCTG
**NDUFB8**	CTCCTTGTTGGGCTTATCACA	GCCCACTCTAGAGGAGCTGA
**SDHB**	AAGCATCCAATACCATGGGG	TCTATCGATGGGACCCAGAC
**UQCRC2**	GTTTGTTCATTAAAGCAGGCAGTAG	TGCTTCAATTCCACGGGTTATC
**ATP5F1A**	ACTGGGCGTGTCTTAAGTATTG	ACCAAGGGCATCAACTACAC
**18S**	CTCAACACGGGAAACCTCAC	CGCTCCACCAACTAAGAACG
**MTCO1**	CCCACCGGCGTCAAAGTAT	TGCAGCAGATCATTTCATATTGC
**B2M**	TGCTGTCTCCATGTTTGATGTATCT	TCTCTGCTCCCCACCTCTAAGT
**HPV16E1**	AACGTGTTGCGATTGGTGTA	TACGCAATTTTGGAGGCTCT
**HPV16E2**	GCCAACACTGGCTGTATCAA	CATCCTGTTGGTGCAGTTAAA
**HPV16E4**	TCCAATGCCATGTAGACGAC	GCTCACACAAAGGACGGATT
**HPV16E5**	CCACAACATTACTGGCGTGC	GCAGAGGCTGCTGTTATCCAC
**HPV16E6**	TCAGGACCCACAGGAGCG	CCTCACGTCGCAGTAACTGTTG
**PPARGC1A**	CCAAGTCGTTCACATCTAGTTCA	TCTGAGTCTGTATGGAGTGACAT
**PPARGC1B**	CCACATCCTACCCAACATCAAG	CACAAGGCCGTTGACTTTTAGA
**ESRRA**	AGGGTTCCTCGGAGACAGAG	TCACAGGATGCCACACCATAG

Supplemental Table 3. Antibodies

Target	Clonality	Immunogen	Isotype	Host	Dilution	Supplier	Catalog#
**PGC1α**	Polyclonal	The C terminal region of human PGC-1α	IgG	Rabbit	1:500	Sigma Aldrich	HPA063136
**PGC1α**	Polyclonal		IgG	Rabbit	1:500	Sigma Aldrich	SAB2106455
**ERRα**	Polyclonal	The N terminal region of human ERRα	IgG	Rabbit	1:1000	Sigma Aldrich	HPA053785
**p53 (1C12)**	Monoclonal	Residues surrounding Ser20 of human p53 protein	IgG1	Mouse	1:1000	Cell Signaling	2524
**p53 (7F5)**	Monoclonal	The amino terminus region of human p53 protein	IgG	Rabbit	1:1000	Cell Signaling	2527
**β-actin (AC-15)**	Monoclonal	The β-cytoplasmic N-terminal sequence	IgG1	Mouse	1:5000	Sigma Aldrich	A3854
**HA (C29F4)**	Monoclonal	HA-Tag	IgG	Rabbit	1:500	Cell Signaling	3724
**OxPhos Human Antibody**<br>Complex I-NDUFB8<br>Complex II-SDHB<br>Complex III-UQCRC2<br>Complex IV-MTCO2P12<br>Complex V- ATP5F1A	Cocktail, monoclonal	Full length protein	IgG	Mouse	1:1000	Thermo Fisher	45-8199

Supplemental Table 4. Plasmids

Plasmid backbone	Insert	Promoter	Tag(s)	Selectable Marker	SOURCE
pLentiN	none	CMV	Met/Flag/HA	Blasticidin	Addgene #37444
pLentiN 16E6no*	HPV16E6 (V42L)	CMV	Flag/HA	Blasticidin	Addgene #37445
pMD2.G	VSV G	CMV			Addgene #12259
psPAX2	Gag-Pro-Pol				Addgene #12260
pcDNA4 myc PGC-1 alpha	PGC-1 alpha	CMV	Myc/His	Zeocin	Addgene # 10974
2kB PGC-1α promoter	Human PGC-1α promoter	pGL3-basic	Luciferase		D. Kelly lab
MSCV-IP N FlagHA 16E6	HPV16 E6 (V42L)	MSCV LTR	Flag/HA	Puromycin	E. White lab Plasmid # 6724
pMSCV-N-HAonly 16E6	HPV16 E6	MSCV LTR	HA	Puromycin	Addgene # 42603
pMSCV-N-HA 16E6 8S9A10T	HPV16 E6 (8S9A10T)	MSCV LTR	HA	Puromycin	Addgene # 44153
pMSCV-N-HA 16E6 I128T	HPV16 E6 (I128T)	MSCV LTR	HA	Puromycin	Addgene # 44154
pMSCV-N-HA 16E6 Star	HPV16 E6 I*	MSCV LTR	HA	Puromycin	E. White lab Plasmid # 7257
